# Establishing knowledge on the sequence arrangement pattern of nucleated protein folding

**DOI:** 10.1371/journal.pone.0173583

**Published:** 2017-03-08

**Authors:** Fei Leng, Chao Xu, Xia-Yu Xia, Xian-Ming Pan

**Affiliations:** Key Laboratory of Bioinformatics, Ministry of Education, School of Life Sciences, Tsinghua University, Beijing, China; Russian Academy of Medical Sciences, RUSSIAN FEDERATION

## Abstract

The heat-tolerance mechanisms of (hyper)thermophilic proteins provide a unique opportunity to investigate the unsolved protein folding problem. In an attempt to determine whether the interval between residues in sequence might play a role in determining thermostability, we constructed a sequence interval-dependent value function to calculate the residue pair frequency. Additionally, we identified a new sequence arrangement pattern, where like-charged residues tend to be adjacently assembled, while unlike-charged residues are distributed over longer intervals, using statistical analysis of a large sequence database. This finding indicated that increasing the intervals between unlike-charged residues can increase protein thermostability, with the arrangement patterns of these charged residues serving as thermodynamically favorable nucleation points for protein folding. Additionally, we identified that the residue pairs K-E, R-E, L-V and V-V involving long sequence intervals play important roles involving increased protein thermostability. This work demonstrated a novel approach for considering sequence intervals as keys to understanding protein folding. Our findings of novel relationships between residue arrangement and protein thermostability can be used in industry and academia to aid the design of thermostable proteins.

## Introduction

(Hyper)thermophilic proteins obtained from (hyper)thermophiles generally remain structurally stable and functionally active at high temperatures [1,2]. Therefore, these proteins provide a unique opportunity to gain understanding of the protein folding problem, the precise mechanisms of which are of long-standing interest in protein science and remains unsolved [3]. Additionally, thermally stable proteins can be used for designing efficient enzymes that remain active at higher temperatures [4–6]. Accordingly, understanding the principles behind thermal stability would be extremely valuable for both theoretical research and industrial applications.

Comparisons between (hyper)thermophilic proteins and their mesophilic homologues have previously been performed, with results indicating that the two typically share 40% to 85% sequence similarity while their three-dimensional (3D) structures are highly superimposable [1,7,8]. Moreover, statistical potentials derived from Boltzmann’s law were developed in recent decades to study protein folding and stability [9–11]. Factors, such as salt bridges, interaction networks, and hydrophobic cores, contribute to changes in the protein free energy during the folding process [12–15]. However, there are no commonly accepted rules linking structure and thermal stability, and occasionally, the rules currently in place tend to be contradictory [16,17].

Efforts have been made to develop approaches that enable modification or design of more satisfactory protein products. These include direct evolution approaches combined with certain diversity-generation and screening or selection methods. However, these methods have their limitations and are both time consuming and cost intensive [18].

In this work, we constructed an equation to describe the impact of sequence intervals between paired residues based on the energy contribution of these pairs to protein folding and analyzed a large sequence dataset available from Swiss-Prot [19] using a novel statistical method. Our statistical analysis showed that in a protein sequence, like-charged residues tend to be adjacently assembled, while unlike-charged residues are distributed over longer sequence intervals. Interestingly, our results indicated that increasing the sequence interval between unlike-charged residues could increase protein thermostability.

## Methods

### Dataset construction

We downloaded 118,848 globular protein sequences with lengths > 60 and known organism optimal growth temperatures (*OGTs*) for of 1211 protein families from the Swiss-Prot database (v2015_10). According to the definition of a protein family, proteins within a given family are likely to have statistically significant sequence similarity (> 30%), whereas proteins between families are unlikely to share statistically significant sequence similarity (< 30%). The dataset used here, included 100,834 mesophilic proteins and 18,014 thermophilic proteins.

Because reported *OGT*s are often given in a temperature range, we repeatedly ran statistics for every protein 11 times at different temperatures, from *OGT* ±5°C, with 1°C increases for each run.

We constructed a new dataset which is randomly shuffled within the sequences. For each N- to C-terminal sequence in the original dataset, each residue was randomly exchanged with others in a randomly selected position in a stepwise process. This procedure was repeated 500 times and generated the new sequences, each having the same amino acid composition, but different amino acid arrangements within the original sequences.

### Sequence-interval-dependent contribution

In a given sequence, a pair value is not counted as one, but rather as a certain contribution coefficient. Similar to previous results, a paired potential will improve along with the increase in its sequence interval. Due to contact restrictions during protein folding, we hypothesize that this increase obeys a sigmoidal law:

$$CC(n)=\frac{1}{1+10.0\text{exp}(-2n)}$$ (1)

where *n* is the sequence interval between two residues, and *CC(n)* is the contribution coefficient of the sequence interval. In order to make sure *CC(n)* is close to zero when n = 0, we add coefficient 10.0 to *exp(-2n)*.

For a pair, *i*, separated by *n* residues in a protein, the contribution is calculated as:

CON(i,n)=num(i,n)*CC(n) (2)

where *CON(i*,*n)* is the contribution of the pair, *i*, separated by *n* residues, *num(i*,*n)* is the number of the pair separated by *n* residues, and *CC(n)* is the contribution coefficient of *n*. The contribution of the pair, *i*, is the sum of all sequence intervals, which is:

$$CON(i)=\sum_{n=0}^{\infty }CON(i,n)$$ (3)

According to Eq (3), contribution of the pair (*CON_random(i)*) in randomly shuffled dataset is also computed. Then, the rectified contribution of the pair (*CON’(i)*) is calculated as:

CON′(i)=CON(i)−CON_random(i) (4)

### Sequence contributions of 210 pairs

For a given protein sequence, the statistical potential was denoted as follows:

$$P=\sum_{i=1}^{210}CON\prime (i)$$ (5)

The relative fraction of the pair contribution, *CF(i)*, was calculated as follows:

$$CF(i)=\frac{CON\prime (i)}{P}$$ (6)

## Results

### Charged residue arrangement

The number of salt bridges or salt bridge networks contribute specifically to changes in protein free energy during the folding process [20–22]. In addition to intra-helical salt bridges, tertiary salt-bridge interactions also play a vital role in thermostability [23]. In our previous work, we reported that the free energy contribution of a salt bridge formed by two charged residues located far apart within the sequence was higher than that of salt bridges formed between two charged residues in close proximity [24]. Also, Tompa et al observed that thermophilic proteins form additional long-range interactions [25]. In this study, the acidic residues, Glu and Asp, were referred to as *A*, and the basic residues, Arg and Lys, were referred to as *B*. The unlike-charged pairs (*AB* salt bridge) represented a favorable pairing with regard to folding, while the like-charged pairs (*AA/BB*) did not favor folding. We counted the number of residue pairs (*N*_*S*_) in a given protein sequence separated by a short interval (*n* < *N*_*S*_), as well as the number of residue pairs (*N*_*L*_) separated by a long interval (*n* > *N*_*L*_). We then used *N*_*S*_ and *N*_*L*_ to calculate the fraction of residue pairs separated within a short interval (*SP*), which is: SP = *N*_*S*_ / (*N*_*S*_ + *N*_*L*_).

**Fig 1:**
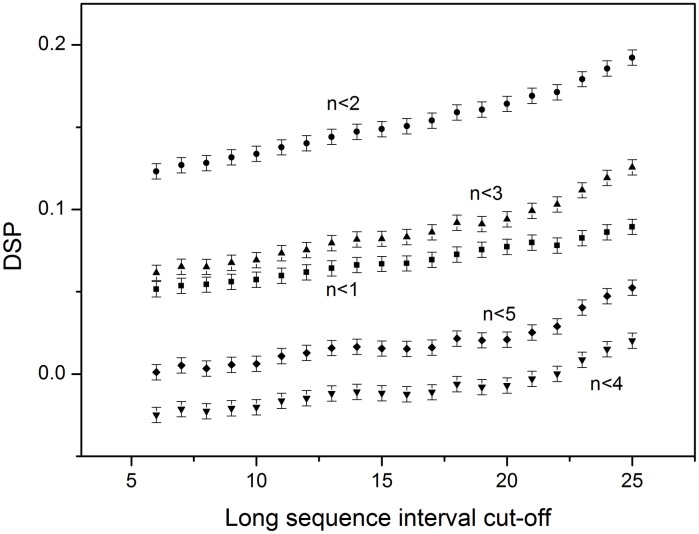
*DSP* against the long-sequence-interval cut-off. Filled squares represent the short-sequence-interval cut-off: *N*_*s*_ = 1 (*n* < 1); filled circle: *N*_*s*_ = 2 (*n* < 2); filled triangle: *N*_*s*_ = 3 (*n* < 3); filled inverted triangle: *N*_*s*_ = 4 (*n* < 4); filled rhombus: *N*_*s*_ = 5 (*n* < 5). Error bars represent a 95% confidence interval. *DSP*, average difference of *SP* in the charged-residue arrangement pattern in a given sequence; *N*_*S*_, residue pairs separated by a short interval.

In previous reports, long range was often defined as separation by more than five amino acids [26]. Accordingly, we set *N*_*S*_ = 1 to 5 and *N*_*L*_ = 6 to 26 as cut-off values for defining the short interval and long interval, respectively. We then first calculate the *SP* values of the *AB* and *AA/BB* pairs and then compare them with the *OGT* over the sequence dataset. Finally, we calculated the average difference of *SP* (*DSP*) between SP of AA/BB and SP of AB. DSP denotes the charged residues arrangement pattern in a given sequence. A value of *DSP* > 0 signified that the fraction of like-charged residue pairs separated by a short interval was larger than that of the fraction of unlike-charged residues. For each *N*_*S*_, we plotted *DSP* against *N*_*L*_ (Fig 1), resulting in the *DSP* values for *N*_*S*_ = 1, 2, 3, and 5 being larger than 0, although when *N*_*S*_ = 2 (*n* < 2 as a short interval), the *DSP* values were the largest. In this study, we set *N*_*S*_ = 2 as the short–sequence-interval cut-off and *N*_*L*_ = 6 as the long-sequence-interval cut-off. In this case, the uncounted fractions (sequence interval between 2 and 5) of the *AB* and *AA/BB* pairs amounted to < 2.3%.

**Fig 2:**
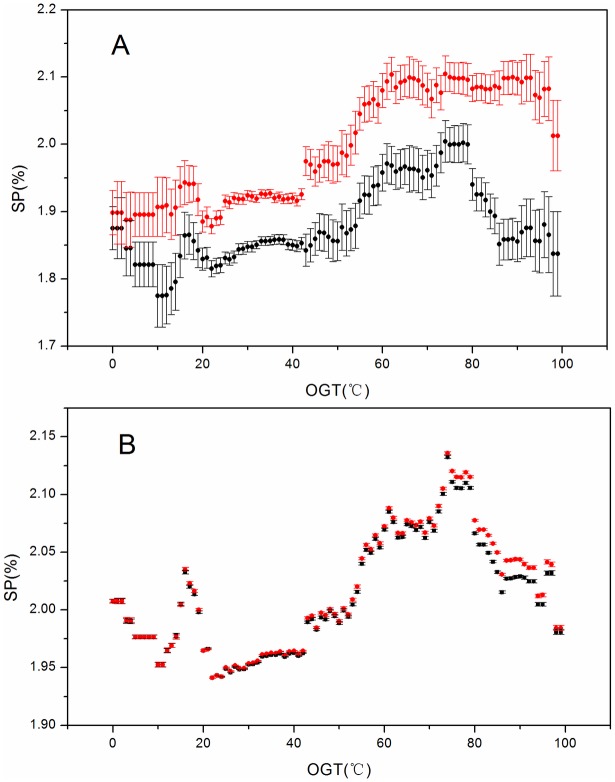
*SP* values against *OGT*. *SP* represents the proportion (%) of short-sequence-interval pairs with cut-off values of *N*_*S*_ = 2 and *N*_*L*_ = 6. (A) Calculated from a raw dataset. (B) Calculated from a random dataset. The black filled symbols correspond to the *AB* pair (unlike-charged residue pairs), and the red-filled symbols correspond to the *AA/BB* pair (like-charged residue pairs). Error bars represent a 95% confidence interval. *A*, Glu and Asp; *B*, Arg and Lys; *N*_*L*_, residue pairs separated by a long interval; *N*_*S*_, residue pairs separated by a short interval; *SP*, the fraction of residue pairs separated within a short interval.

The *SP* results with *N*_*S*_ = 2 and N_*L*_ = 6 are shown in Fig 2A. The values of both *SP(AB)* and *SP(AA/BB)* increased when *OGT* increased from 0°C to 60°C. Interestingly, *SP(AA/BB)* was always larger than *SP(AB)*, implying that in the protein sequence the like-charged residues tended to be adjacently assembled, while the unlike-charged residues were distributed over longer intervals.

Furthermore, when the *OGT* increased from 75°C to 90°C, the value of *SP(AA/BB)* reached a plateau, whereas *SP(AB)* decreased from 75°C to 90°C, implying that at this temperature range, the heat-tolerance mechanisms of (hyper)thermophilic proteins were increased by increasing the sequence interval between unlike-charged residues. When the *OGT* was > 90°C, the value of *SP(AB)* reached a plateau, whereas that of *SP(AA/BB)* decreased, implying that at temperatures > 90°C, other interactions possibly also contributed to heat-tolerance mechanisms [27].

It should be noted that the differences in values between *SP(AB*) and *SP(AA/BB)* were small (~0.2 per 100 residues; Fig 2A). Because (hyper)thermophilic proteins and their mesophilic homologues typically share 40% to 85% sequence similarity, with 3D structures that are highly superimposable, the difference in sequence arrangement between (hyper)thermophilic proteins and their mesophilic homologues should be very small. Additionally, the fold energy of a globular protein is ~1.5 kJ/(mol·K), while the energy of an ion pair is between ~12 kJ/mol and ~21 kJ/mol [28]. Thus, small changes in charged-residue arrangement in a given sequence could confer larger influences on protein thermostability.

For comparative purposes, we calculated the *SP(AB)* and *SP(AA/BB)* values in the randomly shuffled dataset, with results showing that both *SP(AB)* and *SP(AA/BB)* increased beyond the *OGT* from 20°C to 80°C before subsequently decreasing (Fig 2B). Fig 2B shows that *SP(AA/BB)* coincided almost exactly with *SP(AB)*, with no significant difference, indicating that the differences between the original *SP(AB)* and *SP(AA/BB)* were not random.

### Sequence-interval-dependent-pair value

Statistical potentials derived from Boltzmann’s law were developed in recent decades to study protein folding and stability. To reveal the principles behind protein thermal stability, we analyzed the relative frequency of 210 possible residue pairs against the *OGTs*. Having observed that sequence intervals between paired residues was a key to understanding protein folding, we constructed a function (Eq 1) to calculate the contribution coefficient for each sequence interval (*CC(n)*). When n = 0 (when two paired residues are adjacent), the value of *CC(n)* is ~0.09, implying that most of the contact energy of such a pair would not contribute to protein folding, whereas n ≥5, the value of *CC(n)* is > 0.99, implying that the contact energy of such a pair would almost fully contribute to protein folding. Considering that the possibility of generating native conformations from a denatured state is dramatically decreased with increasing sequence separation, *CC(n)* may be regarded as an approximation of the energy contribution of the native conformation to the average energy of denatured states.

### Contribution (CON) of charged pairs versus the OGT

**Fig 3:**
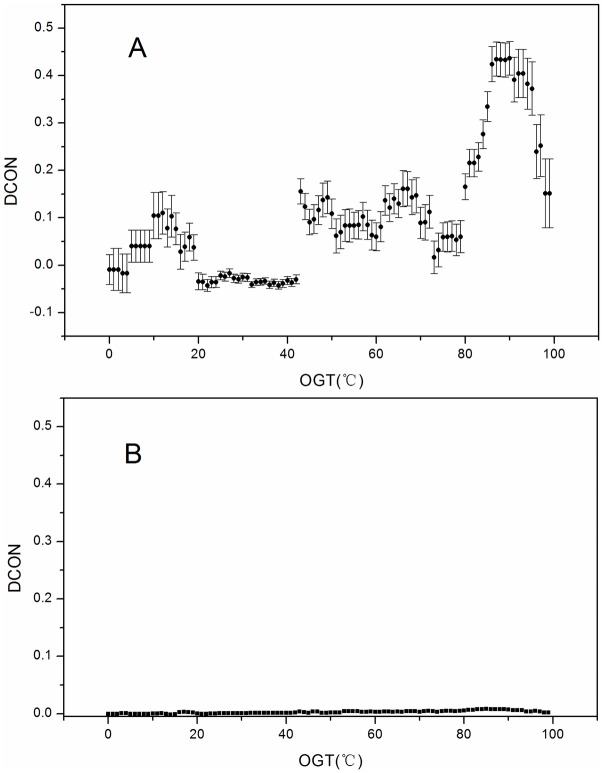
Plot of *DCON* values against the *OGT*. *DCON* is the average *CON*-value difference between the *AB* pair and the *AA/BB* pair. (A) Calculated from a raw dataset. (B) Calculated from a random dataset. Error bars represent the 95% confidence interval. *CON*, contribution; *DCON*, difference in the *CON*; *OGT*, optimal growth temperature.

We calculated the average *CON* of both the unlike-charged pair *AB* (*CON(AB)*) and the like-charged pair *AA/BB* (*CON(AA/BB)*) in proteins having different *OGTs* in the dataset. Furthermore, we also calculated the difference in the *CON* [*DCON* = *CON*(*AA* / *BB*) − *CON*(*AB*)] and plotted it against the *OGT* (Fig 3A). The results of *DCON* against the *OGT* were consistent with those from *SP* against the *OGT* shown in Fig 2A. The value of *DCON* increased along with increases in the *OGT* from 75°C to 90°C and decreased when *OGT* was > 90°C (Fig 3A). Similarly, the value of SP(AB) decreased along with increases in the *OGT* from 75°C to 90°C and SP(AA/BB) decreased when the *OGT* was > 90°C (Fig 2A).

In order to demonstrate the non-random distribution of the *DCON*, we also calculated the *DCON* using the randomly shuffled dataset. As shown in Fig 3B, for any *OGT*, the *DCON* always approximated to zero, which differed from results in Fig 3A.

### Contribution fraction distribution for 210 pairs

As shown in Figs 2A and 3A, the contribution fraction did not exhibit a linear relationship between the *SP* of charged pairs and the temperature. This indicated that protein thermostability was not only the result of interactions between charged residues, but also a consequence of contributions from interactions between other residues. Therefore, it was necessary to investigate the sequence intervals in all 210 pairs.

**Fig 4:**
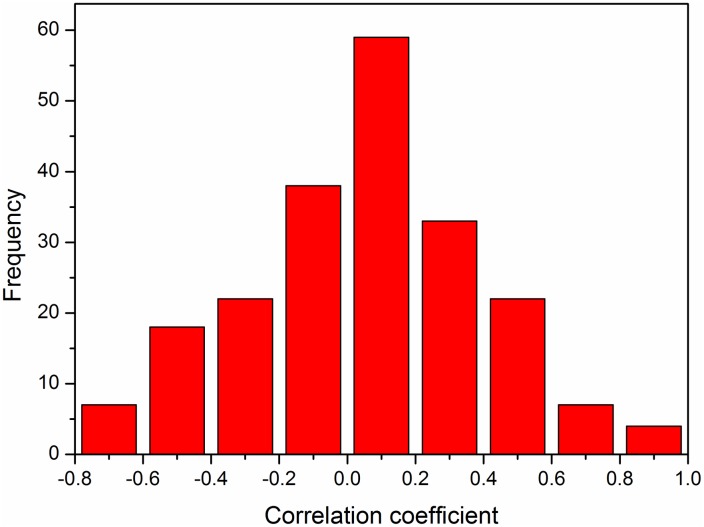
Correlation-coefficient distributions of the 210 pairs at the *OGT*. The X-axis shows the correlation coefficient, and the Y-axis shows the frequency of each correlation-coefficient bin. *OGT*, optimal growth temperature.

We statistically analyzed all sequences in the dataset and calculated the relative fraction of the pair contribution *(CF)* for 210 pairs for each sequence, followed by calculation of the correlation coefficients, slopes, and F-values between the relative frequency of the 210 pairs and the *OGT*s. The correlation-coefficient distribution for the 210 pairs is shown in Fig 4, and it is similar to a normal distribution. Within the 210 pairs, there were 9 pairs with absolute correlation coefficients > 0.7 (whose p-values < 0.05), of which 4 pairs exhibited absolute correlation coefficients > 0.8. S1 Table summarizes all of these results.

**Fig 5:**
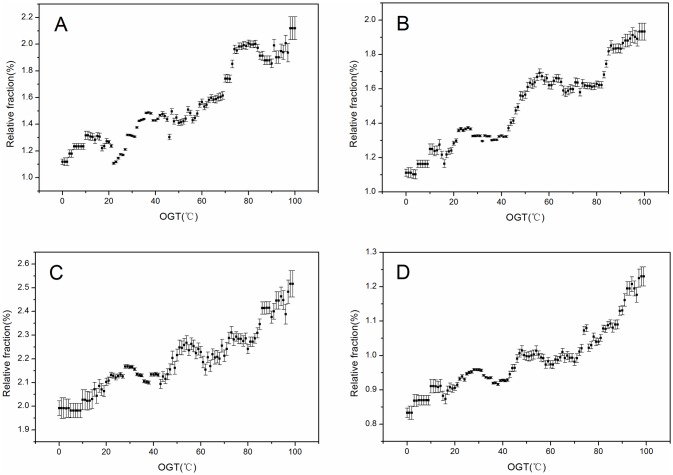
Relative fraction of pairs against the *OGT*. (A) K-E; (B) R-E; (C) L-V; (D) V-V. Error bars represent the 95% confidence interval. *OGT*, optimal growth temperature.

Here, we identified four pairs, the relative fractions of which correlated strongly (absolute correlation coefficient > 0.8) with the *OGTs*. Among these pairs, all contained a charged residue (E, R, or K), or a hydrophobic residue (V, L). These findings were consistent with previous studies on the fraction of amino acids [29]. Fig 5 shows the relative fraction against the *OGTs* of the four pairs, including K-E (correlation coefficient = 0.88, p-value = 0.008), R-E (correlation coefficient = 0.86, p-value = 0.009), L-V (correlation coefficient = 0.85, p-value = 0.01), V-V (correlation coefficient = 0.83, p-value = 0.012). The high values associated with these pairs demonstrated the importance of an interaction, including electrostatic interactions, hydrophobic interactions, and hydrogen bonds, to facilitate accurate *T*_*m*_ predictions as previously reported [30–34].

## Discussion

(Hyper)thermophilic proteins have been studied in terms of folding and function, with the results greatly contributing to protein engineering in the chemical, biotechnological, and food industries. Despite the large number of studies, a comprehensive understanding of factors that determine protein thermal stability remains incomplete. However, protein thermal stability increases with in the presence of *T*_*m*_, suggesting that it would be helpful to investigate factors that closely correlate with *T*_*m*_ values and improve the effectiveness of experimental and computational methods for designing and engineering thermo-stable proteins. Protein function is closely linked with its structural rigidity and flexibility [35], making it necessary to achieve a balance between these characteristics to maintain activity while tolerating temperature increases. Through pluralistic evolution, thermophilic and mesophilic proteins optimized their functions within their respective optimal temperature ranges. In thermophilic organisms, proteins evolved to resist high temperatures, with these proteins generally more rigid than their mesophilic homologues within the same temperature range. However, although mesophilic proteins lose their activity at high temperatures, thermophilic proteins are resistant to high temperatures, even while enduring increases in highly flexibility [36]. No additional stabilizing interactions contribute to the rigidity of thermophilic proteins, which are stabilized by electrostatic and hydrophobic interactions, similar to their mesophilic homologues. However, the residues contributing to these interactions can be conserved in atomic packing configurations while not being absolutely conserved according to sequence alignments [37]. The positions of charged amino acids in thermophilic proteins can be adjusted without notably altering the protein structure. In fact, adjustment of the sequence interval is a convenient means to alter protein stability at different temperatures while retaining similar protein structures between homologous proteins to maintain normal function [38]. Specifically, we find that increasing the sequence interval between unlike-charged residues or decreasing the sequence interval between like-charged residues increased protein *T*_*m*_. This result can be applied in industry and academia to aid in the design of thermostable proteins.

Large amounts of statistical potentials dependent upon spatial structures were derived to gain insight into protein thermostability and used in attempts to explain these mechanisms [9–11]. Although these approaches made progress, they suffered from lack of data, because most protein 3D structures have not been solved. By contrast, there are large numbers of protein sequences, with these numbers continuing to increase. Unlike previous studies, we statistically analyzed the relative frequency of sequence separation between charged-residue pairs versus the *OGTs* derived from a sequence database. Although this approach is not directly related statistically to the relative frequency of contacts between charged-residue pairs in a given protein structure, these should correlate with one another. This implies that a large relative frequency of contacts and in sequence separation between charged residue pairs would result in an increased possibility that these charged-residue pairs could interact within the protein structure. The advantage of sequence-related statistics in this case is that the quantity of sequence data is much larger than that of structural data, thereby allowing a statistical approach in analyzing larger datasets.

In the unfolded state, amino acids remain capable of interacting with one another, with the ensemble of unfolded conformations resulting in inconsistent positions of paired amino acids within the primary sequence. On average, each residue in a disordered conformation might be capable of existing in eight distinct conformations; therefore, when the sequence interval increases from one to five, the number of possible unfolded conformations increases from eight to 32,768. Given that there is only one native conformation, an increase in sequence interval from one to five decreases the possibility of a protein folding into the native conformation from 1/8 to 1/32,768. Protein thermal stability is a function of the energy difference between the native and denatured states. In the folded state, the energy contribution of a contacted pair is determined solely by the nature of the pair itself, whereas in the unfolded state, the energy contribution of the unfolded state is determined by the average of all possible conformations that the contacted pair could reach [39]. Given that larger sequence intervals result in less possibility of generating native conformations from an unfolded state, the free energy of long-range interactions will be larger than those of short-range interactions. As a result, contributions from long-range interactions to protein thermostability increase. Presumably, the probability of interactions between two distant residues within the primary sequence in the unfolded state is very small, whereas the likelihood of possible interactions between paired residues in close proximity to one another is higher. Our findings presented here provided a better understanding of the mechanisms involved in protein folding as a function of thermal stability.

## Supporting information

S1 Table Summarization of the correlation coefficients (R), slopes and P-values. (DOC)
