# Supplementary material for: Establishing knowledge on the sequence arrangement pattern of nucleated protein folding
Source: PLoS One. 2017 Mar 8;12(3):e0173583. doi: 10.1371/journal.pone.0173583 (PMC5342263; doi:10.1371/journal.pone.0173583)
Supplement: S1 Table — (DOC) [file pone.0173583.s001.doc]

**Supplementary Information**

**Table 1** **Summarization of the correlation coefficients (R), slopes and P-values**

| Pairwise | R | Slope(per 100℃) | P-value |
| --- | --- | --- | --- |
| K-E | 0.8807 | 0.0095 | 0.007979 |
| R-E | 0.8614 | 0.0069 | 0.009288 |
| L-V | 0.8449 | 0.0039 | 0.010553 |
| V-V | 0.8291 | 0.0031 | 0.011902 |
| F-Q | 0.7914 | 0.001 | 0.015738 |
| I-V | 0.7834 | 0.0036 | 0.016676 |
| A-V | 0.7656 | 0.0035 | 0.018936 |
| Q-I | 0.691 | 0.0028 | 0.031443 |
| D-L | 0.6842 | 0.0034 | 0.032863 |
| D-T | 0.6154 | 0.0025 | 0.050412 |
| T-N | 0.6093 | 0.0022 | 0.052275 |
| D-A | 0.5998 | 0.0048 | 0.055283 |
| T-A | 0.593 | 0.002 | 0.05752 |
| Y-A | 0.5566 | 0.0011 | 0.070722 |
| C-Q | 0.5559 | 0.0006 | 0.070997 |
| F-L | 0.5418 | 0.0022 | 0.076713 |
| T-T | 0.5306 | 0.0009 | 0.081497 |
| Q-V | 0.5244 | 0.0018 | 0.08424 |
| T-Q | 0.5186 | 0.0019 | 0.086869 |
| A-N | 0.5036 | 0.004 | 0.093951 |
| H-N | 0.4928 | 0.0005 | 0.099308 |
| F-V | 0.4851 | 0.0017 | 0.103261 |
| A-Q | 0.4712 | 0.0021 | 0.110684 |
| H-L | 0.4658 | 0.0009 | 0.113669 |
| G-I | 0.4607 | 0.0013 | 0.11654 |
| Q-G | 0.4382 | 0.0018 | 0.129819 |
| S-A | 0.4347 | 0.0024 | 0.131975 |
| M-V | 0.4249 | 0.0017 | 0.138144 |
| Q-L | 0.4152 | 0.0009 | 0.14444 |
| W-T | 0.4103 | 0.0004 | 0.147694 |
| C-N | 0.4095 | 0.0005 | 0.148229 |
| Y-Q | 0.404 | 0.0007 | 0.151948 |
| D-Q | 0.4023 | 0.0011 | 0.153109 |
| N-L | 0.399 | 0.0027 | 0.155381 |
| H-T | 0.3989 | 0.0007 | 0.15545 |
| D-D | 0.391 | 0.001 | 0.160981 |
| R-G | 0.3876 | 0.0025 | 0.1634 |
| W-Q | 0.3621 | 0.0004 | 0.182292 |
| K-T | 0.3613 | 0.0024 | 0.182905 |
| K-P | 0.3534 | 0.001 | 0.189037 |
| D-S | 0.3417 | 0.0017 | 0.198348 |
| P-P | 0.3398 | 0.0004 | 0.199886 |
| I-M | 0.3281 | 0.0015 | 0.209512 |
| D-C | 0.3278 | 0.0006 | 0.209762 |
| Y-S | 0.3196 | 0.0006 | 0.216673 |
| F-A | 0.3103 | 0.0008 | 0.224668 |
| D-Y | 0.3016 | 0.0007 | 0.232297 |
| A-L | 0.3013 | 0.0033 | 0.232562 |
| W-A | 0.2994 | 0.0005 | 0.234248 |
| D-F | 0.2971 | 0.0006 | 0.236298 |
| S-T | 0.2808 | 0.0012 | 0.251106 |
| F-I | 0.2772 | 0.0019 | 0.254441 |
| S-N | 0.2674 | 0.0008 | 0.263637 |
| F-F | 0.2611 | 0.0007 | 0.269636 |
| F-N | 0.2593 | 0.0009 | 0.271363 |
| R-A | 0.2526 | 0.0013 | 0.277838 |
| L-M | 0.2448 | 0.001 | 0.28547 |
| T-I | 0.239 | 0.0006 | 0.291209 |
| K-Q | 0.2381 | 0.0007 | 0.292105 |
| H-F | 0.2351 | 0.0004 | 0.295098 |
| Y-G | 0.2281 | 0.0005 | 0.302138 |
| H-W | 0.2137 | 0.0001 | 0.316851 |
| A-I | 0.2135 | 0.0029 | 0.317058 |
| F-M | 0.2105 | 0.0004 | 0.320162 |
| T-M | 0.2039 | 0.0003 | 0.327035 |
| N-I | 0.2029 | 0.0018 | 0.328081 |
| H-A | 0.1971 | 0.0004 | 0.334178 |
| A-A | 0.1954 | 0.002 | 0.335973 |
| R-D | 0.1885 | 0.0012 | 0.343298 |
| S-S | 0.1832 | 0.0006 | 0.348965 |
| S-L | 0.1815 | 0.0005 | 0.35079 |
| I-I | 0.1777 | 0.0032 | 0.354882 |
| H-S | 0.1744 | 0.0002 | 0.358449 |
| P-V | 0.1695 | 0.0003 | 0.363768 |
| H-G | 0.1686 | 0.0005 | 0.364748 |
| N-Q | 0.1636 | 0.0005 | 0.370207 |
| R-C | 0.1608 | 0.0001 | 0.373276 |
| D-V | 0.1557 | 0.0008 | 0.378887 |
| E-T | 0.1514 | 0.0004 | 0.383637 |
| K-W | 0.1488 | 0.0002 | 0.386518 |
| H-Q | 0.1395 | 0.0002 | 0.396873 |
| H-M | 0.1386 | 0.0001 | 0.397879 |
| S-Q | 0.1385 | 0.0005 | 0.397991 |
| A-M | 0.1379 | 0.0003 | 0.398662 |
| T-L | 0.1336 | 0.0006 | 0.403481 |
| H-V | 0.1298 | 0.0003 | 0.407751 |
| N-P | 0.1266 | 0.0005 | 0.411356 |
| W-N | 0.1129 | 0.0001 | 0.42687 |
| F-S | 0.1129 | 0.0003 | 0.42687 |
| D-I | 0.1128 | 0.0009 | 0.426984 |
| G-M | 0.1108 | 0.0002 | 0.429259 |
| R-H | 0.109 | 0.0002 | 0.431309 |
| G-L | 0.1035 | 0.0003 | 0.437582 |
| Y-W | 0.1029 | 0.0001 | 0.438268 |
| D-W | 0.1021 | 0.0002 | 0.439182 |
| M-M | 0.0984 | 0.0001 | 0.443414 |
| W-M | 0.0943 | 0.0001 | 0.448111 |
| E-Q | 0.0938 | 0.0004 | 0.448685 |
| F-T | 0.0925 | 0.0002 | 0.450176 |
| K-D | 0.0913 | 0.0007 | 0.451553 |
| Q-P | 0.0882 | 0.0002 | 0.455113 |
| Y-M | 0.0844 | 0.0002 | 0.459482 |
| D-P | 0.0842 | 0.0002 | 0.459712 |
| Y-I | 0.0836 | 0.0002 | 0.460402 |
| W-I | 0.0768 | 0.0002 | 0.468234 |
| Y-P | 0.0627 | 0.0001 | 0.484509 |
| N-G | 0.0611 | 0.0002 | 0.486358 |
| R-N | 0.0602 | 0.0002 | 0.487398 |
| K-G | 0.0522 | 0.0003 | 0.496647 |
| K-C | 0.0465 | 0.0001 | 0.503238 |
| G-G | 0.0459 | 0.0002 | 0.503932 |
| W-G | 0.0413 | 0 | 0.50925 |
| I-L | 0.0352 | 0.0007 | 0.5163 |
| G-P | 0.0328 | 0.0001 | 0.519073 |
| A-P | 0.0315 | 0.0001 | 0.520574 |
| C-G | 0.028 | 0 | 0.524615 |
| S-I | 0.0222 | 0.0001 | 0.531305 |
| N-N | 0.0197 | 0 | 0.534186 |
| H-I | 0.0183 | 0 | 0.535799 |
| W-W | 0.0178 | 0 | 0.536375 |
| W-V | 0.0175 | 0 | 0.53672 |
| W-F | 0.0091 | 0 | 0.54638 |
| N-M | 0.0091 | 0 | 0.54638 |
| E-C | 0.0076 | 0 | 0.548103 |
| Y-V | 0.0027 | 0 | 0.553723 |
| Y-F | -0.0003 | 0 | 0.557158 |
| S-V | -0.0132 | 0 | 0.571879 |
| W-S | -0.0186 | 0 | 0.578013 |
| C-S | -0.0256 | 0 | 0.585935 |
| N-V | -0.0273 | -0.0001 | 0.587854 |
| E-L | -0.0369 | -0.0003 | 0.59865 |
| D-H | -0.037 | -0.0001 | 0.598762 |
| D-N | -0.041 | -0.0001 | 0.603239 |
| K-H | -0.0428 | -0.0001 | 0.605249 |
| Y-N | -0.0506 | -0.0001 | 0.613926 |
| Y-L | -0.0575 | -0.0001 | 0.621554 |
| C-P | -0.0631 | 0 | 0.627711 |
| D-M | -0.0722 | -0.0001 | 0.637646 |
| E-N | -0.0939 | -0.0003 | 0.660956 |
| Q-M | -0.0961 | -0.0002 | 0.663286 |
| W-P | -0.0977 | -0.0001 | 0.664978 |
| K-S | -0.1053 | -0.0006 | 0.672965 |
| T-V | -0.1116 | -0.0005 | 0.679527 |
| D-E | -0.1126 | -0.0008 | 0.680564 |
| Y-C | -0.1157 | -0.0002 | 0.683768 |
| S-G | -0.1181 | -0.0009 | 0.686239 |
| Y-Y | -0.1201 | -0.0001 | 0.688292 |
| R-T | -0.1233 | -0.0004 | 0.691565 |
| W-L | -0.1325 | -0.0002 | 0.700888 |
| S-P | -0.1478 | -0.0003 | 0.716103 |
| H-P | -0.149 | -0.0002 | 0.717281 |
| R-Q | -0.1501 | -0.0004 | 0.718358 |
| S-M | -0.154 | -0.0004 | 0.722162 |
| K-L | -0.1593 | -0.0012 | 0.72729 |
| R-I | -0.1679 | -0.0006 | 0.73551 |
| C-I | -0.1725 | -0.0008 | 0.739855 |
| C-M | -0.1733 | -0.0001 | 0.740607 |
| H-H | -0.1761 | -0.0003 | 0.743229 |
| I-P | -0.1769 | -0.0005 | 0.743976 |
| E-G | -0.1831 | -0.001 | 0.749725 |
| T-P | -0.189 | -0.0006 | 0.755131 |
| E-A | -0.1941 | -0.0014 | 0.759753 |
| F-G | -0.1953 | -0.0004 | 0.760833 |
| D-G | -0.2045 | -0.0009 | 0.769029 |
| C-V | -0.2065 | -0.0011 | 0.770789 |
| Y-T | -0.2084 | -0.0004 | 0.772455 |
| E-H | -0.2096 | -0.0006 | 0.773504 |
| R-P | -0.2243 | -0.0005 | 0.786124 |
| T-G | -0.2287 | -0.0012 | 0.78982 |
| K-K | -0.24 | -0.0013 | 0.799139 |
| G-V | -0.2427 | -0.0009 | 0.801329 |
| H-Y | -0.2433 | -0.0002 | 0.801813 |
| F-P | -0.2651 | -0.0005 | 0.818932 |
| E-S | -0.2826 | -0.0012 | 0.831982 |
| C-T | -0.2959 | -0.0004 | 0.841485 |
| C-A | -0.3101 | -0.0005 | 0.851234 |
| H-C | -0.315 | -0.0003 | 0.854503 |
| R-K | -0.3234 | -0.0015 | 0.859993 |
| F-C | -0.3249 | -0.001 | 0.860959 |
| K-A | -0.3278 | -0.0017 | 0.862813 |
| C-L | -0.3502 | -0.0012 | 0.876562 |
| K-F | -0.3521 | -0.0024 | 0.877682 |
| A-G | -0.3581 | -0.002 | 0.881173 |
| C-C | -0.3724 | -0.0016 | 0.889206 |
| E-W | -0.3797 | -0.0011 | 0.893154 |
| L-L | -0.4104 | -0.0077 | 0.908646 |
| L-P | -0.411 | -0.0014 | 0.908931 |
| R-S | -0.422 | -0.0011 | 0.914044 |
| K-Y | -0.4277 | -0.0011 | 0.916608 |
| R-W | -0.4278 | -0.0011 | 0.916652 |
| W-C | -0.4475 | -0.0003 | 0.925066 |
| E-I | -0.4948 | -0.0049 | 0.942614 |
| R-M | -0.501 | -0.001 | 0.944652 |
| R-L | -0.5052 | -0.0025 | 0.946 |
| R-R | -0.5089 | -0.0037 | 0.947166 |
| K-I | -0.5238 | -0.005 | 0.951659 |
| E-P | -0.525 | -0.0016 | 0.952007 |
| M-P | -0.5327 | -0.0008 | 0.954194 |
| R-Y | -0.5376 | -0.0011 | 0.955543 |
| E-F | -0.5773 | -0.0025 | 0.965333 |
| E-Y | -0.5774 | -0.0021 | 0.965355 |
| E-M | -0.5902 | -0.0018 | 0.968103 |
| R-V | -0.596 | -0.0035 | 0.969288 |
| K-N | -0.6101 | -0.0031 | 0.972017 |
| R-F | -0.6249 | -0.0015 | 0.974661 |
| E-E | -0.6491 | -0.0034 | 0.978533 |
| K-V | -0.6696 | -0.0044 | 0.981409 |
| K-M | -0.683 | -0.0024 | 0.983107 |
| E-V | -0.7021 | -0.0073 | 0.985296 |
| Q-Q | -0.7053 | -0.0016 | 0.985638 |
